# Studies on 16*α*-Hydroxylation of Steroid Molecules and Regioselective Binding Mode in Homology-Modeled Cytochrome P450-2C11

**DOI:** 10.1155/2011/918168

**Published:** 2010-07-27

**Authors:** Hamed I. Ali, Morio Yamada, Yukihisa Fujita, Mitsuko Maeda, Eiichi Akaho

**Affiliations:** ^1^Faculty of Pharmacy, Helwan University, Ain Helwan, Cairo 11795, Egypt; ^2^Department of Chemistry, Hyogo College of Medicine, 1-1, Mukogawa-cho, Nishinomiya, Hyogo 663-8501, Japan; ^3^Liberal Arts Center, Hyogo University of Health Sciences, 1-3-6, Minatojima, Chuo-ku, Kobe 650-8530, Japan; ^4^Faculty of Pharmaceutical Sciences, Center for Area Research and Development (CARD), Kobe Gakuin University, 1-1-3, Minatojima, Chuo-ku, Kobe 650-8586, Japan

## Abstract

We investigated the 16*α*-hydroxylation of steroid molecules and regioselective binding mode in homology-modeled cytochrome P450-2C11 to correlate the biological study with the computational molecular modeling. It revealed that there was a positive relationship between the observed inhibitory potencies and the binding free energies. Docking of steroid molecules into this homology-modeled CYP2C11 indicated that 16*α*-hydroxylation is favored with steroidal molecules possessing the following components, (1) a bent A-B ring configuration (5*β*-reduced), (2) C-3 *α*-hydroxyl group, (3) C-17*β*-acetyl group, and (4) methyl group at both the C-18 and C-19. These respective steroid components requirements were defined as the inhibitory contribution factor. Overall studies of the male rat CYP2C11 metabolism revealed that the above-mentioned steroid components requirements were essential to induce an effective inhibition of [^3^H]progesterone 16*α*-hydroxylation. As far as docking of homology-modeled CYP2C11 against investigated steroids is concerned, they are docked at the active site superimposed with flurbiprofen. It was also found that the distance between heme iron and C16*α*-H was between 4 to 6 Å and that the related angle was in the range of 180 ± 45°.

## 1. Introduction

Cytochrome P450 (P450) constitutes a large superfamily of heme-containing enzymes capable of oxidizing a variety of substrates, both of endogenous (such as steroids) and exogenous (xenobiotics) origins [1–7]. Although a variety of P450s are able to metabolize a broad range of substrates, the enzymes often exhibit strict regio- and stereoselectivity towards pertinent compounds, such as various steroids [1]. One of the most active and versatile P450 is rat CYP2C11, a microsomal P450 isoform catalyzing more than 90% of steroid 16*α*-hydroxylations [8–10]. It is well-known that several 3-keto-4-ene steroids such as progesterone and testosterone are metabolized in a gender-specific and predominant manner by the adult rat liver microsomes. In the male, these steroids are primarily metabolized into two oxidized (16*α*-hydroxyl and 6*β*-hydroxyl) products mainly by the respective, male-specific cytochrome P450 subforms, CYP2C11 and CYP3A2, while they are primarily metabolized into the 5*α*-reduced products by female predominant 5*α*-reductase [11]. Most of P450 structures reveal that the heme group is buried deep within the protein matrix, indicating that residues outside of the active site may also be required to guide the substrate into the heme pocket by recognizing substrates at the protein surface and/or comprising part of a substrate access channel [12]. 

In recent years, homology modeling has become an important tool to study the P450 function, especially in conjunction with experimental approaches [13]. A large amount of work has been directed to elucidating the substrate-binding sites of various P450s, and the understanding of this field is now becoming increasingly important, mainly using the two powerful techniques, site-directed mutagenesis and computational molecular modeling of the relevant P450s [11, 12, 14–16]. In homology modeling, a 3-dimensional (3D) model of the protein is constructed based on its amino acid sequence and on the crystal structure of one or more reference proteins. This mainly involves a sequence alignment between the protein and the template(s) [17].

A challenge remains still for the development of a precise 3D-crystal structure of CYP2C11. Therefore, in this study, an investigation was carried out on the docking mode of 71 different steroid molecules against a computationally homology-modeled 3D-structure of CYP2C11, so as to see a correlation of the biologically obtained results with the AutoDock computational results.

## 2. Results and Discussion

### 2.1. Homology Modeling of CYP2C11

Homology modeling of CYP2C11 was performed by the use of Swiss-Model software [13, 18]. The amino acid sequence of CYP2C11 structure was used as target protein. Various proteins of 500 residues were used as templates, including P450 2C9 with warfarin bound, PDB code, log5 [19]; P450 2C9, PDB code, log2 [19]; P450 2C9 complexed with flurbiprofen bound, PDB code, 1r9o [20]; P450 2C8, PDB code, 1pq2 [21]; P450 2C5/3LVdH complexed with a bound substrate, 4-methyl-N-methyl-N-(2-phenyl-2H-pyrazol-3-yl)benzenesulfonamide (DMZ), PDB code, 1n6b [13]; P450 2C5/3LVdH complexed with diclofenac, PDB code, 1nr6 [14]; P450 2C5, PDB code, ldt6 [15]; P450 2B4 with 4-(4-Chlorophenyl) imidazole bound, PDB code, 1suo [22]; P450 2B4, PDB code, 1po5 [22]; P450 2A6 with methoxsalen bound, PDB code, 1zll [23]; P450 2A6 with coumarin bound, PDB code, 1z10 [17]; P450 3A4, PDB code, 1tqn [24]; P450 3A4 with metyrapone bound, PDB code, 1w0g [25]; P450 3A4, with PDB code, 1w0e [25]; P450 3A4 with progesterone bound, PDB code, 1w0f [20]; CYP51 with estriol bound, PDB code, 1x8v [26]; CYP51 in ferric low spin state, PDB code, 1h5z [20]; C37L/C151T/C442A-triplet mutant of CYP51, PDB code, lu13 [27]; CYP51 with 4-phenylimidazole bound, PDB code, 1e9x [27]; CYP51 with fluconazole bound, PDB code, 1eal [27]. 

Mammalian CYP450 proteins recognize and metabolize diverse xenobiotics such as drug molecules, environmental compounds, and pollutants. Human CYP450 proteins, CYP1A2, CYP2C9, CYP2C19, CYP2D6, and CYP3A3 are the major drug—metabolizing isoforms, and contribute to the oxidative metabolism of more than 90% of the drugs in current clinical use [20]. Therefore, the organism for which any of our P450 structures/models study originate from are mostly human [13–15, 18–27]. 

The pairwise sequence alignments of the target sequence with that of template was carried out and the sequence identity of templates with the target sequence is shown in Table 1. The amino acid sequence of the aligned protein templates of chain A of P450 2C9-flurbiprofen (1r9o), P450 2C9-warfarin (log5), and P450 2C9 (log2) exhibited the highest percentage of identity with that of CYP2C11 in the range of 83.5%, 75.9%, and 75.9%, respectively. The chain A of the templates of CYP51-estriol (1x8vA), CYP51 (1h5zA), C37L/C151T/C442A (1u13A), CYP51-4-phenylimidazole (1e9xA), and CYP51-fluconazole (1ea1A), whose percentages of identity were 23.3%, 23.9%, 23.9%, 23.9%, and 23.9%, respectively, had been rejected due to their too low similarities with the target sequence. 

### 2.2. Docking of Representative Steroids in the Embedded Flurbiprofen Pocket

Seventy one different steroid molecules (1–71) (Table 2) were docked in the 16*α*-hydroxylation orientation into the biding site of the homology-modeled CYP2C11, where the ligand, flurbiprofen (FLP), was embedded. Affinity orientation between the protein and the substrate are predominately hydrophobic. The side chains of Asn107, Ile113, Phe114, Asn204, Phe205, Phe208, Phe237, Thr292, Asp293, Gly296, Ala297, Glu300, Thr301, and Leu366 lay within 4Å of all docked steroid molecules as pointed out in Figure 2, where Gly296, Ala297, and Leu366 are hidden for clarity. Seven of these amino acids, namely Phe114, Asn204, Asp293, Gly296, Ala 297, Thr301, and Leu366 corresponded identically to the key amino acid residues identified in the earlier studies of the binding site of flurbiprofen in CYP2C9 (PDB code, 1r9o) [20], as cited in PDP sum, http://www.ebi.ac.uk/pdbsum/, accessed on November 12, 2008.

**Figure 1:**
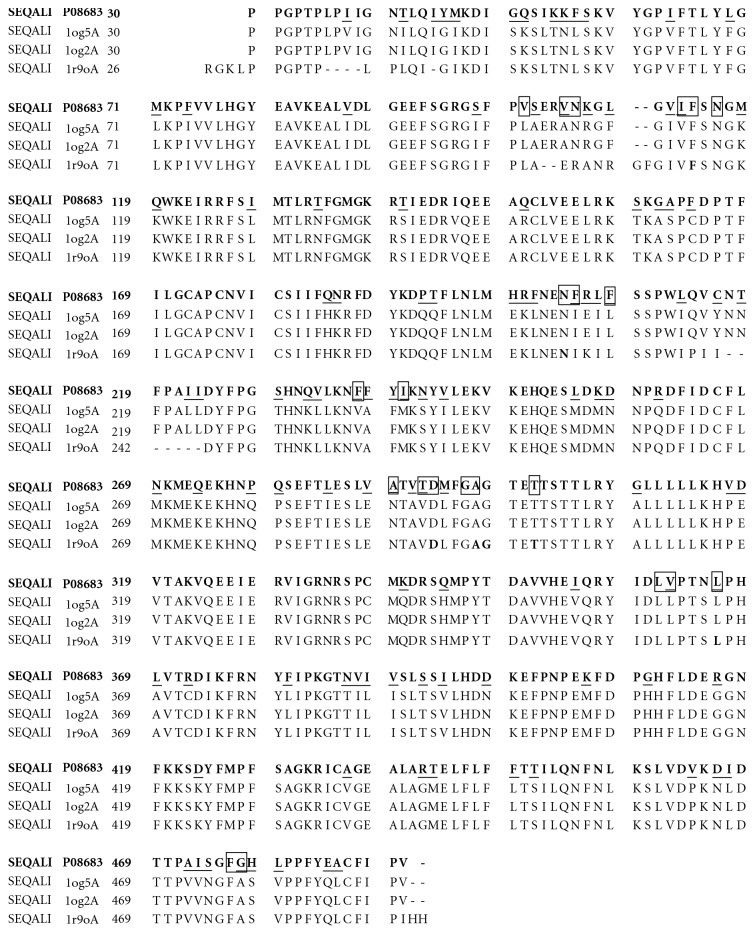
The sequence alignment among CYP2C11(Swiss-prot entry code: P08683), and chain A of warfarin-bound CYP2C9 (PDB code:1og5), CYP2C9 (PDB code:1og2), flurbiprofen-bound CYP2C9 (PDB code:1r9o). The first 29, 29, 29, and 25 amino acids of these proteins, respectively, are not shown and were not modeled. Residues of target CYP2C11 are highlighted in bold letters, the amino acids of the binding site are indicated by boxed text, the nonmatched amino acids are underlined, the identical key amino acid residues of 1rgo with that of CYP2C11 are shown in bold Arial black letters, and the amino acid sequences of chain B of 1og5 and 1og2 were deleted due to their identical amino acid composition to their chain A.

**Figure 2:**
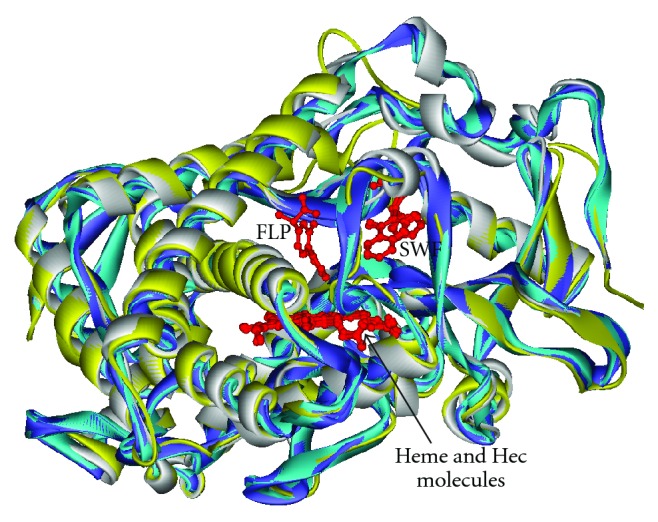
The Homology-modeled structure of CYP2C11, in white solid ribon in sequence alignment with the solid ribon crystal structures of CYP2C9 (1og5) in cyan with its bound ligandSWF(s-warfarin), CYP2C9 (1og2) in violet, and CYP2C9 (1r9o) in yellow with its bound ligand, FLP (Flurbiprofen). Molecules of heme and Hec of the aligned proteins in red ball and stick are shown in exact superposition within the binding site.

The amino acid sequence of the aligned protein templates of chain A of lr9o.pdb, log5.pdb, and log2.pdb exhibited the highest percentage of identity with that of CYP2C11 in the range of 83.5%, 75.9%, and 75.9%, respectively, as shown in Figure 1 and Table 1. The above two findings strongly support the hypothesis that the key amino acid residues of CYP2C11 are identical, for the most part, to that of CYP2C9. However, this finding must be further verified experimentally. Figure 2 illustrates the ribbon schematic presentations of the homology model of CYP2C11 in sequence alignment with the warfarin—bound CYP2C9 (PDB code, log5) [19], with CYP2C9 (PDB code, log2) [19], and with the flurbiprofen-bound CYP2C9 (PDB code, 1r9o). The details of these sequence views are shown in Figure 1, including both the proposed key amino acid residues and the different and similar residues of the aligned protein sequences. 

### 2.3. Hydroxylation of Steroids and Their Docking Conformation within the Active Site

The ideal conformation of the steroid molecules within their binding site in varieties of P450s was proposed by many investigator [28–33]. They reported that the respective substrates for prokaryotic P450s cam, and eryF are positioned in such a way that a substrate is hydroxylated at a distance of 4.5 and 4.8 Å from the heme Fe to the hydroxylated atom [34]. These substrates were also oriented in such a way that the hydrogen, which is abstracted during the reaction, be located within 2 Å of the oxygen of the oxy-preferryl intermediate [13]. It is also reported that the docked substrates should be located with the distance between their oxidation site (C16) and the heme iron being 6 Å and with the C-H-Fe angle at C16 being 180° [28]. The C-H bond in C-H-Fe sequence should be perpendicular to the heme surface. The substrate was usually placed at a position equivalent to that of camphor in the P450cam crystallographic structure, which gives a distance of about 4.2–4.9 Å between the oxidation sites and the heme iron. However, molecular dynamics simulations of camphor-bound P450cam suggests that the average distance between the carbon atom, at which hydroxylation takes place, and the heme iron is 5.3 Å.

Szklarz et al. [8] proposed that for catalysis to occur the following conditions must be met: (1) the distance between the hem iron and the carbon, at which the hydroxylation takes place, must be 5.6–6 Å to allow room for the active oxygen, which results in the carbon to active oxygen distance of 3.9–4.2 Å, and the hydrogen to oxygen distance of 2.3–3.1 Å and (2) the angle between the carbon, the hydrogen, and the heme iron (or active oxygen) should be close to 180° (180 ± 45°) to promote hydrogen bond formation. Therefore, the analysis of our docking study revealed that the results met the above-mentioned requirements for catalysis, (1) and (2) proposed by Szklarz et al. [8]. That is, the binding orientation would place a potential site for C-16*α*-hydroxylation within 5-6 Å of the heme iron and the angle between the carbon, the hydrogen and the heme iron (or active oxygen) should be as close as possible to 180° (180 ± 45°).

Analysis of the docking results revealed that there were a considerable number of conformations flexibilities of the docked substrates oriented in order to meet the above-mentioned conditions, and it was noticed that many conformations were docked within the required distance (4–6 Å), but not by the required angle (180 ± 45°).

### 2.4. The Docking Energy of Binding and the Experimentally Observed Inhibitory Potency

Inhibitor docking studies revealed that there was a reasonable positive relationship between their observed inhibitory potencies against [3H]PROG16*α*-hydroxylation and the number of conformations met with the above mentioned condition (Table 2). In this type of a comparative study between biological potency and computational simulation, it is of our primary concern whether the correlation coefficient is positive or not. In order to examine this relationship, the correlation between the AutoDock inhibition constant (Ki) of steroid substrates and the inhibition potency (IC_40_) against [^3^H]progesterone 16*α*-hydroxylation of the rat liver microsome was plotted. As shown is Figure 5, the correlation coefficient was positive and it showed that our model and described enzymatic mechanism were valid. 

### 2.5. Docking Mode of the Most Potent Inhibitor, 3*α*-Hydroxy-5*β*-Pregnan-20-One (33)

**Figure 3:**
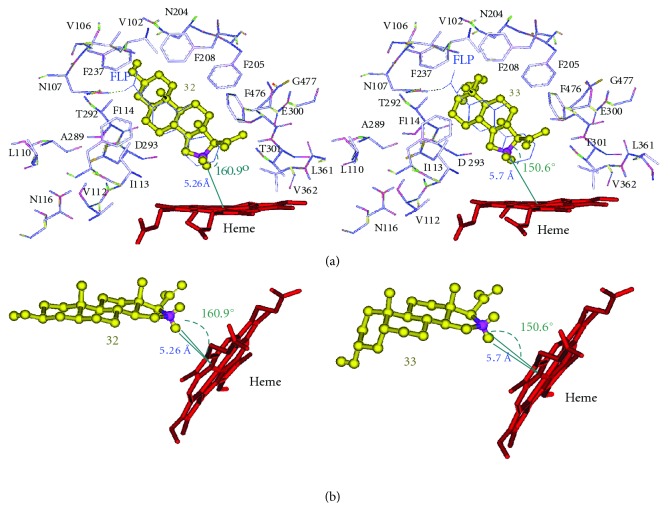
Docking configurations of inhibitors 32 and 33 (yellow ball and stick) into the homology-modeled CYP2C11 where amino acid residues G296, A297, and L366 are hidden for clarity. (a) The relevant amino acid (wire, colored by atoms) biding sites with the inhibitors are shown, and both inhibitors are docked in a superimposed fashion with the embedded substrate flurbiprofen (FLP, wire in blue) within RMSD of 0.93 and 0.79 Å, respectively. (b) Inhibitor 32 exhibits the planar A-B ring binding configuration with heme molecule (stick in red), while inhibitor 33, the bent A-B ring binding configuration. The distance between C16-carbon of inhibitor 32 and the heme iron is 5.26 Å with the angle between C16 carbon, C16-*α* hydrogen, and the heme iron being 160.9°, while the formaer of inhibitor 33, 5.70 Å, and the latter, 150.6°.

**Figure 4:**
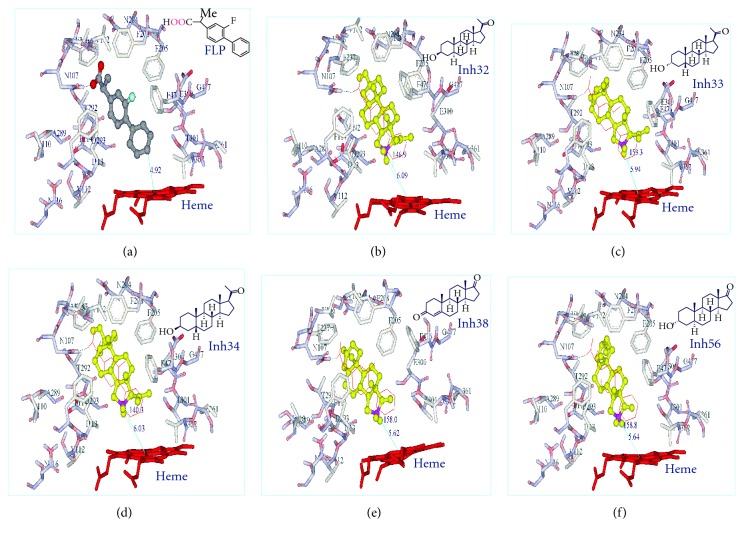
The docking modes of different inhibitors, inh32, inh33, inh34, and inh56 (inhibitor code), are shown with ball and stick in yellow within the binding site pocket of Cyt P450-2C11. The embedded ligand, flurbiprofen (FLP, ball and stick colored by atoms) is bound inside the pocket of the homology-modeled Cyt P450-2C11. All inhibitors are docked within the distance of 4–6 Å (shown in lines and 3 digit numbers) between C16 and the iron atom of heme molecule (shown as red, stick) and with the angel between the 16C*α*-hydrogen and the iron of heme molecule being 180 + 45° (shown in lines and 4 digit numbers). Pertinent amino acids (ASN107, ILE113, PHE114, ASN204 PHE205, PHE237 THR292, ASP293, GLY296, ALA297, GLU300, THR301, and LEU366) lay within 4 Å of all docked steroid molecules and are shown in stick, colored by atoms. Hydrogen bond formation is shown in dotted line between the inhibitor and amino the acid residue.

**Figure 5:**
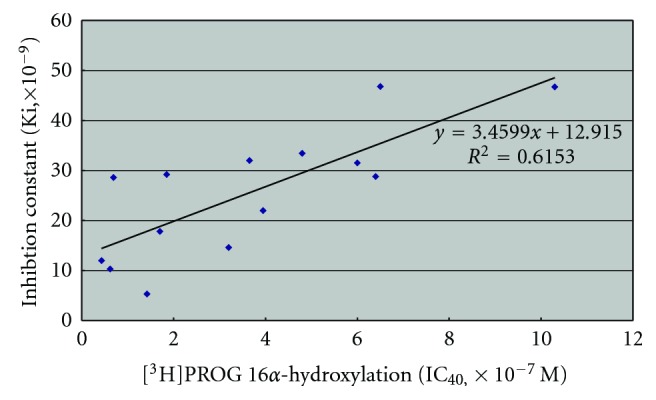
Correlation relationship between the AutoDock inhibition constant (Ki) of tested steroid substrates and inhibition potencies (IC_50_) against [^3^H]PROG 16*α*-hydroxylation by rat liver microsomes- three outsider points were eliminated.

The steroid molecule 3*α*-hydroxy-5*β*-pregnan-20-one (33), as shown in Table 2, exhibited the highest number of the conformations met with the above-mentioned conditions with the lowest binding free energy (∆Gb) of −10.09 kcal/mol, and the minimum inhibition constant (Ki) of 4.03 × 10^−8^, that is, with the highest binding affinity (IC_40_; = 0.24 × 10^−7^ M) within the CYP2C11 binding site pocket. The docked inhibitor 33, as shown in Figure 4, was located within 5.7 Å between the C16-carbon atom, where the proposed 16*α*-hydroxylation takes place, and the heme iron, and the angle between C16-carbon, C16-*α*-hydrogen, and the heme iron was 150.6°. The RMSD (distance in Å, measured between the centeroid of the docked substrate and that of the bound ligand, flurbiprofen) was 1.03 Å. Also, the inhibitor 33 showed a bent A-B ring configuration within the binding site pocket, as shown in Figure 3(b). 

### 2.6. Docking Modes of the Other Pertinent Steroid Molecules: 1, 29, 32, 34, 38, and 56

Docking of other inhibitors, namely 1, 29, 32, 34, 38, and 56, met the condition requirement exhibiting the favorable distance and angle of their sites of oxidation and the heme iron. They are positioned so that their C16-carbon atoms be located within 5.73, 5.82, 5.26, 5.59, 5.53, and 5.51 Å from the heme iron, respectively, and their angles between C16-carbon, C16-*α*-hydrogen, and the heme iron were 144.8°, 148.9°, 160.9°, 146.7°, 156.1°, and 153.1°, respectively. Their corresponding RMSD were 0.92, 1.07, 0.73, 0.71, 0.82, and 4.65 Å, respectively. Thus, it was noticed that these substrates were docked exactly in the same position within the binding site pocket and they seem to superimpose with the bound ligand, flurbiprofen, as their RMSD distances are quite small with the average of 1.48 Å. Out of the above six inhibitors, Figures 3 and 4 illustrate the actual docking mode of inhibitors 32, 33, 34, 38, and 56.

Inhibitors 1 and 38 with 4-ene A-B ring and inhibitors 29, and 56 with 5*α*-reduced A-B ring are shown in Figure 3 with planar A-B ring configuration, whereas inhibitor 34 with 5*β*-reduced A-B ring exhibited bent A-B ring configuration within the binding site. 

## 3. Conclusion

Computer simulated automated docking studies were performed using AutoDock 3.05. Docking results revealed that there was a variety of conformations of the docked inhibitors meeting the confirmation of the reported orientation requirements of steroids within their binding sites [7, 13, 21]. The docked inhibitors were shown to be positioned so that the site of hydroxylation (C16-carbon) resides within 5-6 Å from the heme iron, which is consistent with the distances seen in the case of other P450 substrate complex, with the angle between C16-carbon, C16*α*- hydrogen, and the heme iron being 180 ± 45.0°. It was noticed that steroids were docked exactly overlapped with the flurbiprofen, as their average RMSD was 1.98 Å. Also a positive correlation was obtained between the observed inhibitory potencies against [^3^H]PROG 16*α*-hydroxylation and the binding free energies of the docked steroids. The correlation between the observed inhibitory potencies and AutoDock inhibition constants (ki), exhibited also a positive correlation coefficient. Steroid molecule 33 exhibited the lowest binding free energy, that is, the highest affinity within the binding site of CYP2C11, and with the highest number of conformations meeting the reported requirements. This agrees well with the biologically observed results; its observed inhibitory potency index against [^3^H]PROG 16*α*-hydroxylation was 31.46 (IC_40_;: 3*α*-hydroxy-5*β*-pregnan-20-one 33, 0.24 × 10^−7^ M, vs. progesterone 1, 7.55 × 10^−7^ M).

 As a whole, the results of the present docking investigation revealed that many amino acid residues responsible for binding of the flurbiprofen-bound CYP2C9 (1r9o), were also essential for the interaction between CYP2C11 and inhibitors. Moreover, docking of steroid molecules within the 3-D homology model of CYP2C11 based on that of warfarin-bound CYP2C9 (log5), CYP2C9 (log2), and flurbiprofen-bound CYP2C9 (log5), were in a fair agreement with the observed biological data.

## 4. Methods

### 4.1. Experimental Procedures

#### 4.1.1. Materials

[1,2-3H]Progesterone (PROG) (specific activity, 49.2 Ci/mmol) and [9,11,12-3H]3*α*-OH-5*α*-P(specific activity, 65.0 Ci/mmol) were obtained from PerkinElmer life Sciences, U.S.A. and purified by paper chromatographic system of hexane and saturated formamide (H/F). Unlabeled steroids were purchased from Sigma Chemical Company, St. Louis, Mo, U.S.A., and Steraloids, Inc., Wilton, N.H., U.S.A. Whatman No.1 filter papers used for paper chromatographies were obtained from Whatman Ltd., England. Other reagents were of analytical grade.

### 4.2. Preparation of Adult Male Rat Liver Microsomes

Approximately 95-day-old male Wistar rats, castrated on the 70th day after birth, were used. The liver microsomes were prepared as previously described [29, 31]. The experiments were performed according to instrumental guidelines for the care and use of laboratory animals.

### 4.3. [3H]PROG Metabolism by Rat Liver Microsomes—Inhibitory Effects of Various Unlabeled Steroids

 The metabolism by rat liver microsomes were examined, according to our previously described procedure [23–25]. Briefly, the microsomal suspension (400–600 *μ*g of protein/2.2 mL, total volume of the reaction mixture) was preincubated with [3H]PROG (20 nM) under the absence or presence of an unlabeled steroid (0.01–10 *μ*M) at 36°C for 30 min. Then NADH (3.16 *μ*M) was added, and the reaction mixture was incubated further for 5 min. After the incubation, two identical samples were mixed and extracted with toluene. The toluene-extractable [3H]PROG metabolites (more than 90%) were isolated by various paper chromatographic systems and then identified by recrysallization method [26]. Other miscellaneous procedures are described in our previous papers [29, 31]. 

### 4.4. Protein Homology Modeling

 Since the crystal structure of CYP2C11 is not available, the three dimensional (3D) model of CYP2C11 used in the present simulation was constructed based on a homology modeling method. The homology modeling procedure and the sequence alignment were performed with the cooperation of Swiss-Model (Swiss-Model version 36.0003) [17, 18]. Comparative modeling techniques were used to prepare homology model of CYP 2C11. Several homologous crystal structures were referred to as template structures. The amino acid sequence for the desired protein was referred to as the target. The crystal template structures were selected from ExPDB template database to identify suitable template structures for the comparative modeling. The following templates of 500 sequences residues were downloaded from Brookhaven PDB (http://rcsb.org/pdb/): CYP2C9 with warfarin bound, PDB code, log5; CYP2C9, PDB code, log2; CYP2C9 complexed with flurbiprofen bound, PDB code, 1r9o; CYP2C8, PDB code, 1pq2; CYP2C5/3LVdH complexed with a bound substrate, 4-methyl-N-methyl-N-(2-phenyl-2H-pyrazol-3-yl)benzenesulfonamide (DMZ), PDB code, 1n6b; CYP2C5/3LVdH complexed with diclofenac, PDB code, 1nr6; CYP2C5, PDB code, 1dt6; CYP2B4 with 4-(4-Chlorophenyl) imidazole bound, PDB code, 1suo; CYP2B4, PDB code, 1po5; CYP2A6 with methoxsalen bound, PDB code, lzll; CYP2A6 with coumarin bound, PDB code, 1zl0; CYP3A4, PDB code, 1tqn; CYP3A4 with progesterone bound, PDB code, 1w0f; CYP3A4 with metyrapone bound, PDB code, 1w0g; CYP3A4, PDB code, 1w0e; CYP51 with estriol bound, PDB code, 1x8v; CYP51 in ferric low spin state, PDB code, 1h5z; C37L/C151T/C442A-triplet mutant of CYP51, PDB code, 1ul3; CYP51 with 4-phenylimidazole bound, PDB code, 1e9x; CYP51 with fluconazole bound, PDB code, 1ea1. The target sequence was downloaded from the SWISS-PROT database (http://us.expasy.org/sprot/) (accession number P08683). Running pairwise alignments of the target sequence with that of the template were carried out and the sequence identity of templates with the target is shown in Table 1. Those templates of 1x8vA.pdb, 1h5zA.pdb, lu13A.pdb, 1e9xA.pdb, and 1ea1A.pdb, whose percentages of identity were 23.3%, 23.9%, 23.9%, 23.9%, and 23.9%, respectively, had been rejected due to their too low similarities with the target sequence. The sequence alignment was followed by adding the missing side chains, adding hydrogens, and optimizing loops and OXT (nb = 1); and the final total energy was—17460.258 KJ/mol, and then hydrogens were finally removed.

### 4.5. Automated Docking

 Computer-simulated automated docking studies were performed using the widely distributed molecular docking software, AutoDock 3.05, a grid-based docking program [33], which was utilized for the study of binding mode of inhibitors within CYP2C11. This program addresses automatically the flexible docking of the ligands into a known protein structure. In contract, flexibility of the target protein is not taken into account. 

 AutoDock 3.05 scans the active site for low energy binding models and for suitable orientations of the probe molecule, using a modified genetic algorism that employs a local search (GALS) and precomputed grids for the evaluation of the interaction energy. The target homology-modeled protein CYP2C11 was separated alone by using DS modeling 1.1 software (DS modeling 1.1; Accelrys inc., San Diego, CA (2003)) and representative amino acids of the ligand-binding site were selected within 5 Å neighborhood surrounding the embedded ligand, flurbiprofen. A 120 Å 120 Å 120 Å grid size (x, y, z) with a spacing of 0.300 Å centered at—18.44, 86.67, and 30.89 Å that encompassed the active site where the ligand, flurbiprofen, was embedded, was used to guide the docked inhibitors. The results of 250 randomly seeded runs were analyzed for each of the docked inhibitors. The docked inhibitors were assigned to a cluster if the atomic coordinates of the docked inhibitors exhibited a root-mean-square deviation (RMSD) of less than 0.5 Å difference from each other (RMSD-tolerance of 0.5 Å). The clusters were ranked from the averaged lowest energy obtained for members of the cluster to the highest. The analysis was carried out for the top 10 docking clusters. Each of the clusters that exhibited significant negative interaction energies was examined by DS modeling program to determine their binding orientations.

### 4.6. Preparation of Small Molecules

ChemDraw ultra 8.0 software (Chemical Structure Drawing Standard; Cambridge Soft Corporation, USA(2003)) was used for construction of compounds which were converted to 3D structures using Chem 3D ultra 8.0 software (Molecular Modeling and Analysis; Cambridge Soft Corporation, USA(2003)) and the constructed 3D structures were energetically minimized by using MOPAC with 100 iterations and minimum RMS gradient of 0.10.

### 4.7. Evaluation of Docked Results

 DS modeling 1.7 was utilized for the molecular modeling and the evaluation of H-bonds in ligand-receptor interaction and for the measurement of RMSD, which was computed and expressed in angstrom (Å) as a locational comparison of two relevant molecules of interest. In the actual sense, it was measured as a distance between the centroid of the docked inhibitor and the bound-ligand, flurbiprofen (FLP).

## Figures and Tables

**Table 1:** Percentage of sequence identity of the pairwise alignment of various template sequences with the target protein sequence (CYP2C11).

Crystal structures	PDB code and chain^c^	% identity	Crystal structures	PDB code	% identity
CYP2C9-flurbiprofen	1r9oA	83.46	CYP2A6-methoxsalen	1z11D	52.7
CYP2C9-warfarin	1og5A	75.9	CYP2A6-coumarin	1z10A	52.6
CYP2C9	1og2A	75.9	CYP2A6-coumarin	1z10B	52.3
CYP2C9-warfarin	1og5B	75.9	CYP2A6-coumarin	1z10C	52.7
CYP2C9	1og2B	75.9	CYP2A6-coumarin	1z10D	52.7
CYP2C8	1pq2A	74.3	CYP3A4	1tqnA	28.02
CYP2C8	1pq2B	74.3	CYP3A4-progesterone	1w0fA	30.05
CYP2C5/3LVdH-DMZ^a^	1n6b	73.95	CYP3A4-metyrapone	1w0gA	34.25
CYP2C5/3LVdH-diclofenac	1nr6A	73.95	CYP3A4	1w0eA	28.2
CYP2C5	1dt6A	71.96	CYP51-Estriol	1x8vA	23.33
CYP2B4-CPZ^b^	1suoA	55.1	CYP51	1h5zA	23.9
CYP2B4	1po5A	54.9	C37L/C151T/C442A-triplet mutant of CYP51	1u13A	23.9
CYP2A6-methoxsalen	1z11A	52.2	CYP51-4-phenylimidazole	1e9xA	23.9
CYP2A6-methoxsalen	1z11B	52.6	CYP51-fluconazole	1ea1A	23.9
CYP2A6-methoxsalen	1z11C	52.7

^a^DMZ: 4-Methyl-*N*-methyl-*N*-(2-phenyl-2*H*-pyrazol-3-yl)benzenesulfonamide.

^b^CPZ: 4-(4-Chlorophenyl)-imidazole.

^c^The first four symbols represent PDB code and the last symbol, A, B, C, or D, represents the amino acid chain involved in sequence alignments.

**Table 2:** The inhibitory potency of various steroids on [3H] PROG 16*α*-hydroxylating activity by male rat liver microsomes and their AutoDock results including the binding free energy, the inhibition constant, the distance between C16 carbon atom and feme iron and the angle between C16 carbon atom, C16*α*-hydrogen, and heme Fe iron.

Steroids		[^3^H]PROG16*α*-hydroxylation (IC_40_×10^−7^ M)^a^	∆*G*_*b*_ (kcal/mol)	*Inhibition constant *(*Ki*, x10^−8^)	Distance (Å)	Angle (∘)

No.	Trivial name (°)
(A) 4-Pregnene steroids						

(1)	Progesterone	7.55	−10.93	0.976	5.73	144.8
(2)	3*β*-Hydroxyprogesterone	2.55	—^b^	—	—	—
(3)	6*β*-Hydroxyprogesterone	>10 *μ*M^c^	—	—	—	—
(4)	6*β*-Acetoxyprogesterone	7.40	—	—	—	—
(5)	11*α*-Hydroxyprogesterone	>10 *μ*M	—	—	—	—
(6)	11*α*-Acetoxyprogesterone	>10 *μ*M	—	—	—	—
(7)	11*β*-Hydroxyprogesterone	>10 *μ*M	−11.32	0.504	5.90	139.3
(8)	16*α*-Hydroxyprogesterone	>10 *μ*M	—	—	—	—
(9)	16*α*-Methylprogesterone	>10 *μ*M ^d^	−9.97	4.88	5.32	159.1
(10)	18-Hydroxyprogesterone	>10 *μ*M	—	—	—	—
(11)	19-Hydroxyprogesterone	>10 *μ*M	—	—	—	—
(12)	19-Norprogesterone	>10 *μ*M	—	—	—	—
(13)	20*α*-Hydroxyprogesterone	>10 *μ*M	—	—	—	—
(14)	21-Hydroxyprogesterone	>10 *μ*M	−10.56	1.83	4.73	139.7
(15)	21-Acetoxyprogesterone	>10 *μ*M	—	—	—	—
(16)	Corticosterone	>10 *μ*M	−10.54	1.87	4.75	143.6

(B) 5-Pregnene steroids and cholesterol						

(17)	Pregnenolone	1.42	−11.29	0.532	5.76	139.2
(18)	Pregnenolone-3-acetate	8.00	−9.52	10.6	4.92	166.8
(19)	Pregnenolone-3-sulfate	1.95	—	—	—	—
(20)	5-Pregnene-3,20-dione	>10 *μ*M	—	—	—	—
(21)	20*α*-Hydroxypregnenolone	>10 *μ*M	−10.61	1.67	5.90	134.8
(22)	21-Hydroxypregnenolone	4.62	−10.86	1.09	5.95	144.8
(23)	21-Acetoxypregnenolone	6.00	−12.20	0.114	4.98	136.3
(24)	21-Sulfatepregnenolone	>10 *μ*M	−9.69	7.89	4.53	139.7
(25)	5-Pregnen-3*β*-ol	>10 *μ*M	—	—	—	—
(26)	Cholesterol	>10 *μ*M	−9.79	6.65	6.0	159.2

(C) 5*α*-or 5*β*-Pregnane steroids						

(27)	5*α*-Pregnan-3,20-dione	7.20	−9.51	10.6	5.86	149.1
(28)	5*β*-Pregnan-3,20-dione	3.95	−10.45	2.20	4.93	135.0
(29)	3*α*-Hydroxy-5 *α* -pregnan-20-one	0.62	−10.90	1.03	5.82	148.9
(30)	3*α*-Acetoxy-5 *α* -pregnan-20-one	4.10	—	—	—	—
(31)	3*α*-Sulfate-5 *α* -pregnan-20-one ^c^	>10 *μ*M	—	—	—	—
(32)	3*β*-Hydroxy-5 *α* -pregnan-20-one	2.25	−9.54	10.1	5.26	160.9
(33)	3*α*-Hydroxy-5 *β* -pregnan-20-one	0.24	−10.09	4.03	5.70	150.6
(34)	3*β*-Hydroxy-5 *β* -pregnan-20-one	1.70	−10.57	1.78	5.59	146.7
(35)	3*α*,11*β*-Dihydroxy-5*α*-pregnan-20-one	1.50	—	—	—	—
(36)	3*β*,16*α*-Dihydroxy-5*α*-pregnan-20-one	11.5	—	—	—	—
(37)	3*β*-Pregnan-3-one	>10 *μ*M	—	—	—	

(D) 4-Androstene steroids						

(38)	4-Androsten-3,17-dione	10.0	−11.13	0.693	5.53	156.1
(39)	4-Androsten-3-one; 17-*β*-carboxylaic acid	>10 *μ*M^e^	—	—	—	—
(40)	4-Androsten-3-one;17-*β*-carboxylaic acid methyl ester	8.01	—	—	—	—
(41)	Testosterone	14.5	−10.02	4.49	4.34	151.5
(42)	17*β*-Acetoxytestosterone	>10 *μ*M	—	—	—	—
(E) 5-Androstene steroids						

(43)	Dehydroepiandrosterone	6.80	—	—	—	—
(44)	Dehydroepiandrosterone-3-sulfate	>10 *μ*M	−12.91	0.0346	4.06	140.9
(45)	5-Androstenediol	6.40	−10.29	2.88	4.24	160.1

(F) 5*α*- or 5*β*−-Androstane steroids						

(46)	5*α*-Androstane	>10 *μ*M	−9.69	7.94	5.90	136.4
(47)	5*β*-Androstane	>10 *μ*M	−9.66	8.26	5.94	149.8
(48)	5*α*-Androstan-3*α*-ol	10.3	−10.00	4.67	6.0	139.5
(49)	5*α*-Androstan-3*β*-ol	6.5	−10.58	1.75	4.0	139.2
(50)	5*β*-Androstan-3*α*-ol	5.10	−9.50	10.8	4.55	145.3
(51)	5*β*-Androstan-3*β*-ol	1.60	−9.93	5.30	4.49	165.7
(52)	5*α*-Androstan-17*β*-ol	>10 *μ*M	—	—	—	—
(53)	5*β*-Androstan-17*β*-ol	3.65	−10.22	3.20	5.87	150.6
(54)	5*α*-Androstan-3,17-dione	6.50	—	—	—	—
(55)	5*β*-Androstan-3,17-dione	4.80	−9.98	4.80	5.67	155.2
(56)	3*α*-Hydroxy-5*α*-Androstan-17-one	6.35	−9.69	7.89	5.51	153.1
(57)	3*β*-Hydroxy-5*α*-Androstan-17-one	4.80	−10.20	3.34	4.69	160.9
(58)	3*α*-Hydroxy-5*β*-Androstan-17-one	6.50	−10.00	4.68	5.64	155.4
(59)	3*β*-Hydroxy-5*β*-Androstan-17-one	1.85	−10.28	2.92	4.12	158.0
(60)	5*α*-Dihydrotestosterone	11.5	—	—	—	—
(61)	5*β*-Dihydrotestosterone	6.00	−10.23	3.15	5.64	151.1
(62)	5*α*-Androstan-3*α*,17*β*-diol	1.25	—	—	—	—
(63)	5*α*-Androstan-3*α*,17*β*-diol-17-acetate	3.00	—	—	—	—
(64)	5*α*-Androstan-3*α*,17*β*-diol-17-sulfate^c^	>10 *μ*M	−10.37	2.51	5.45	145.8
(65)	5*α*-Androstan-3*β*,17*β*-diol	11.1	—	—	—	—
(66)	5*β*-Androstan-3 *α*,17 *β* -diol	0.69	−10.29	2.86	5.82	150.0
(67)	5*β*-Androstan-3*α*-ol-17*β*-carboxylic acid	3.60	−11.87	0.201	5.0	138.3
(68)	5*β*-Androstan-3*α*-ol-1*β*-carboxylic acid methyl ester	0.43	−10.81	1.20	5.47	156.0
(69)	5*β*-Androstan-3*β*,17*β*-diol	3.20	−10.69	1.46	4.14	148.8

(G) Estogens						

(70)	Estradiol-17*β*	>10 *μ*M	—	—	—	—
(71)	Estradiol-17*α*	>10 *μ*M	−9.75	7.11	4.15	135.1

^a^IC_40 _value was defined as the molar concentration (×10^−7^ M) of an unlabeled steroid causing 40% inhibition of [^3^H]PROG16*α*-hydroxylation.

^b^Docking results are not matched with the required parameters (distance = 4–6 Å and angle = 180 ± 45°).

^c, e^These imply the mean values of % inhibition ^c, d^IC_40_ value (×10^−7^ M) obtained from extrapolation, and % increase  ^e^, respectively, at 10 *μ*M of the relevant unlabeled compound.
